# Update in Perioperative Ischemic Workup: Integrating 2024 AHA/ACC Guidelines and Contemporary Evidence

**DOI:** 10.3390/jcdd13070309

**Published:** 2026-07-06

**Authors:** Nicholas Mangano, Vanathi Ganesan, Yusef Shibly, Ashley Yu, Meng Wang, Sergio D. Bergese

**Affiliations:** 1 Department of Anesthesiology, Stony Brook University Hospital, Stony Brook, NY 11794, USAmeng.wang@stonybrookmedicine.edu (M.W.); 2 Renaissance School of Medicine, Stony Brook University, Stony Brook, NY 11794, USA; ashley.yu@stonybrookmedicine.edu

**Keywords:** perioperative ischemia, myocardial injury after noncardiac surgery (MINS), preoperative cardiac evaluation, risk stratification, AHA/ACC guidelines, cardiac biomarkers, noncardiac surgery, perioperative cardiovascular risk

## Abstract

Perioperative myocardial ischemia and myocardial injury after noncardiac surgery (MINS) remain prevalent contributors to postoperative morbidity and mortality. Recent advances, including high-sensitivity biomarkers and updated 2024 American Heart Association/American College of Cardiology (AHA/ACC) guidelines, have modified the clinical approach to preoperative ischemic evaluation. This review intends to synthesize contemporary evidence and provide a framework for perioperative ischemic workup. A narrative review of the current literature and major society guidelines was conducted, focusing on perioperative risk stratification, functional capacity assessment, biomarker utilization, noninvasive and invasive diagnostic modalities, and perioperative medical optimization strategies. Contemporary perioperative evaluation favors a stepwise, risk-based approach that uses clinical risk indices, functional capacity, and selective diagnostic testing. Biomarkers such as natriuretic peptides and cardiac troponins enhance risk prediction and enable the detection of MINS, which is strongly associated with increased mortality. Evidence does not support routine preoperative stress testing or prophylactic coronary revascularization in stable patients. Guideline-directed medical therapy, including sustained statin use and attentive management of antiplatelet and beta-blocker therapy, remains central to risk mitigation. Modern perioperative ischemic workup prioritizes individualized, evidence-based evaluation over routine testing. Integration of biomarkers, structured risk assessment, and multidisciplinary management may improve outcomes, though additional research is needed to define optimal strategies for detecting and treating MINS.

## 1. Introduction

### 1.1. Clinical Importance of Perioperative Ischemic Events

Cardiovascular complications after noncardiac surgery are significant causes of postoperative morbidity and mortality. Despite improvements in operative techniques, perioperative monitoring, and perioperative care pathways, myocardial ischemia and infarction continue to occur in a substantial proportion of patients, particularly those with preexisting cardiovascular disease or multiple medical comorbidities. Epidemiologic studies estimate that more than 200 million noncardiac surgical procedures are performed annually worldwide, of which 10 million patients experience perioperative cardiovascular complications within 30 days of their surgeries [1].

Perioperative myocardial ischemia most often results from an imbalance between myocardial oxygen supply and demand. Surgical stress, sympathetic activation, tachycardia, hypotension, anemia, and inflammatory responses may either reduce coronary perfusion or raise myocardial oxygen demand during the perioperative period. These physiologic changes can precipitate ischemia in patients with obstructive coronary artery disease (CAD) or vulnerable plaques [2]. Perioperative plaque rupture and thrombosis can also trigger acute coronary syndromes after surgery, further raising the risk of adverse cardiac events.

In recent years, attention has increasingly focused on the concept of perioperative myocardial injury, with the diagnosis of MINS developed in 2014. MINS was defined by the authors as myocardial injury identified by postoperative elevation in cardiac troponin levels attributable to ischemia within 30 days of surgery, often occurring in the absence of typical ischemic symptoms [3,4]. The Vascular Events in Noncardiac Surgery Cohort Evaluation (VISION) study, a large multicenter prospective trial, demonstrated that postoperative troponin elevations are common and strongly associated with increased short- and long-term mortality following noncardiac surgery [3]. Importantly, most cases of MINS occur silently, without classic clinical manifestations of myocardial infarction, which underscored the limitations of symptom-based diagnostic criteria in the perioperative setting.

The recognition of MINS has modified the understanding of perioperative cardiac risk; rather than emphasizing solely on clinically apparent myocardial infarction, contemporary perioperative medicine must now recognize a wider range of myocardial injury and its associated adverse outcomes. Additional investigations using high-sensitivity cardiac troponin assays have confirmed the prognostic significance of perioperative myocardial injury and strengthened the importance of systematic perioperative cardiovascular risk assessment [5,6].

Effective preoperative ischemic workup is therefore necessary to identify patients at elevated cardiovascular risk prior to noncardiac surgery in order to minimize the risk of MINS. Appropriate evaluation enables clinicians to optimize medical therapy, determine whether additional diagnostic testing is warranted, and develop perioperative management strategies to mitigate ischemic complications. However, indiscriminate testing may delay surgery, raise the healthcare costs, and expose patients to unnecessary procedures without improving outcomes. Consequently, modern perioperative assessment involves a targeted, stepwise approach to cardiovascular risk stratification and diagnostic testing, as emphasized in contemporary clinical practice guidelines [7,8].

The overarching goal of perioperative cardiovascular evaluation is not simply to diagnose coronary artery disease but rather to determine whether additional testing or interventions would meaningfully alter perioperative management or improve clinical outcomes. Achieving this equilibrium between prudent evaluation and avoidance of low-value testing is a key challenge in perioperative medicine.

### 1.2. Historical Perspective

Approaches to perioperative ischemic evaluation have evolved substantially over the past several decades. Early efforts to predict perioperative cardiac risk relied primarily on clinical assessment and physician judgment. One of the earliest systematic attempts to quantify perioperative cardiac risk was the Goldman Cardiac Risk Index, introduced in 1977. This model identified several predictors of postoperative cardiac complications, including a recent history of myocardial infarction, arrhythmias, and signs of heart failure [9].

Later refinements in risk prediction led to the development of the Revised Cardiac Risk Index (RCRI), proposed by Lee and colleagues in 1999. The RCRI simplified risk stratification through incorporating six easily identifiable clinical predictors: ischemic heart disease, heart failure, cerebrovascular disease, insulin-dependent diabetes mellitus, renal dysfunction, and high-risk surgical procedures [10]. Because of its simplicity and reproducibility, the RCRI became widely adopted in clinical practice and remains an important component of perioperative cardiac risk assessment.

During the same period, diagnostic modalities for detecting myocardial ischemia expanded considerably. Noninvasive stress testing, including exercise electrocardiography, pharmacologic stress echocardiography, and nuclear myocardial perfusion imaging, became increasingly utilized to identify inducible ischemia in patients undergoing preoperative cardiovascular evaluation. However, clinical data demonstrated that routine preoperative testing often failed to improve perioperative outcomes unless it led to important changes in management. Population-based studies suggested that widespread use of preoperative stress testing may not significantly improve surgical outcomes when applied indiscriminately [11].

Furthermore, the Coronary Artery Revascularization Prophylaxis (CARP) trial demonstrated that prophylactic coronary revascularization prior to major vascular surgery did not reduce perioperative myocardial infarction or mortality in most patients with stable coronary artery disease [12]. This evidence prompted a shift away from routine testing toward selective diagnostic evaluation based on clinical risk and functional capacity.

Over time, professional organizations developed comprehensive clinical practice guidelines to standardize perioperative cardiovascular evaluation. The 2014 AHA/ACC guidelines on perioperative cardiovascular evaluation provided a commonly used stepwise algorithm uniting clinical risk assessment, surgical risk, and functional capacity to guide further testing [13]. Similar recommendations have been incorporated into international guidelines, including those from the European Society of Cardiology and the Canadian Cardiovascular Society [14,15].

The recently published 2024 AHA/ACC guidelines for perioperative cardiovascular management for noncardiac surgery represent the most up-to-date synthesis of evidence in this field [7]. The guidelines prioritize individualized patient assessment, judicious use of diagnostic testing, and multidisciplinary cooperation between anesthesiologists, cardiologists, and surgeons in choosing optimal perioperative management strategies [7,8].

### 1.3. Purpose & Scope of This Review

Given the continued evolution of perioperative cardiovascular risk assessment and the expanding literature surrounding myocardial injury after noncardiac surgery, an updated synthesis of the current evidence is warranted. Advances in cardiac imaging, increasing use of high-sensitivity cardiac biomarkers, and updated guideline recommendations have all contributed to changes in contemporary approaches to perioperative ischemic evaluation.

The purpose of this review is to supply a comprehensive update on the perioperative ischemic workup for patients undergoing noncardiac surgery, with particular emphasis on recommendations from the 2024 AHA/ACC guidelines and relevant recent literature. Specifically, this manuscript aims to summarize contemporary strategies for clinical risk stratification, including widely used risk indices and the evaluation of functional capacity. In addition, the review will examine how surgical risk and patient comorbidities influence decisions regarding further cardiovascular testing.

Furthermore, this manuscript will discuss the indications, advantages, and limitations of available diagnostic modalities used in preoperative ischemic evaluation, including stress testing, echocardiography, nuclear imaging, and coronary computed tomography. The developing role of perioperative biomarkers (including cardiac troponin and natriuretic peptides) in identifying myocardial injury and guiding perioperative management will also be examined [5,16].

Finally, this review will highlight emerging directions in perioperative ischemic evaluation, including improvements in risk prediction models, incorporation of precision medicine approaches, and the potential use of artificial intelligence-driven clinical decision support tools. As surgical populations continue to age and the prevalence of cardiovascular disease increases, optimizing strategies for identifying and managing perioperative myocardial ischemia will persist as a critical component of improving surgical outcomes.

### 1.4. Methods

A narrative review of the literature was conducted to evaluate contemporary evidence regarding perioperative myocardial ischemia, MINS, and strategies for perioperative ischemic evaluation and management. A comprehensive search of PubMed was performed using combinations of keywords including perioperative myocardial ischemia, MINS, perioperative cardiac risk assessment, functional capacity, stress testing, cardiac biomarkers, natriuretic peptides, troponin, coronary revascularization, and perioperative medical therapy. Additional searches were performed on ClinicalTrials.gov to identify completed, ongoing, and recently published clinical trials relevant to perioperative cardiac risk stratification and management. Reference lists of key publications and guideline documents were also reviewed to identify additional pertinent studies.

Priority was given to randomized controlled trials, systematic reviews, meta-analyses, and major observational studies published in English, as well as current recommendations from professional societies, including the 2024 AHA/ACC Guideline for Perioperative Cardiovascular Evaluation and Management of Patients Undergoing Noncardiac Surgery. Evidence was synthesized narratively, with emphasis on risk stratification, functional capacity assessment, biomarker utilization, diagnostic testing, perioperative optimization strategies, and the detection and management of MINS.

## 2. Risk Stratification in the Perioperative Period

### 2.1. Clinical Risk Scores

Optimizing patient care is important for ensuring postoperative safety, and over the years, perioperative risk calculators (see Table 1) have been developed to improve risk discrimination. Commonly used calculators include the RCRI, the American College of Surgeons National Surgical Quality Improvement Program (NSQIP), Surgical Outcome Risk Tool (SORT), Surgical Risk Scale, and the Gupta Postoperative Respiratory Failure, which are some measures that may be used for qualifying a patient’s readiness for surgery [17].

Patients have an increased risk of cardiovascular disease with age and with the presence of other comorbidities [18,19]. With over 300 million surgeries conducted globally and an aging population [14,20], it is important to utilize the most accurate and appropriate assessment tools. For instance, NSQIP has been shown to improve risk stratification for neurosurgical procedures, and RCRI has mixed data, being more discriminative in abdominal and vascular surgeries in patients aged 65 and older, and in endovascular abdominal aortic aneurysm surgeries [14].

Furthermore, not all risk calculators account for a person’s dynamic health status. The major indices listed above do not include biomarkers that would assess the critical inflammatory state or the body’s response to other stressors during preoperative evaluation [14]. Taking these factors into account is important when considering the type of surgery, its urgency, and the patient’s ability to withstand the procedure’s stress. See Table 1 for a brief overview of different perioperative risk calculators.

### 2.2. Functional Capacity

Assessing the body under stress is another level of evaluation of the cardiopulmonary system. Normally, the functional capacity is a balance of the cardiac, vascular, and pulmonary reserves in response to stress, along with adequate hemoglobin concentration and skeletal muscle function [21,22]. It can be independently associated with mortality and has greater discrimination power in predicting postoperative risk [18,23,24]. This is measured using metabolic equivalents (METs), where one MET represents a resting oxygen consumption of 3.5 mL O_2_/kg/min. Moderate functional capacity is defined as greater than or equal to 4 METs, and the higher the METs, the less likely postoperative complications [25].

One well-known risk assessment tool in perioperative evaluation is the Duke Activity Status Index (DASI), which uses a 12-item questionnaire to quantify patients’ activity levels. Higher scores identify a decreased risk of myocardial injury or infarction, and other postoperative complications [20,26].

The gold standard in assessing functional capacity is cardiopulmonary exercise testing (CPET), where the patient exercises in an interval graded manner as inspired, expired gases, heart rate, and electrocardiogram measurements are obtained [27,28]. It is recommended for those with a forced expiratory volume of less than 60% [28]. One study showed that by analyzing patient METs alongside the DASI risk tool, a DASI score of 34 (on a total DASI range of 0 to 58) was identified as a threshold for predicting postoperative complications, creating an objective measure to supplement the patient interview [23]. While DASI scores are not directly comparable to MET scores, with numerous and often conflicting conversion calculators described in the literature, this DASI threshold score of 34 clinically corresponds with 5 METs when related to peak oxygen consumption (higher than the moderate functional capacity MET threshold of 4) [23]. Newer directions in advancing CPET assessment involve combining it with stress echocardiography: this allows for better visualization of the pathophysiologic mechanisms underlying exercise intolerance in high-risk patients and can lead to customized treatment that targets the root cause of dyspnea and cardiac instability [21].

### 2.3. Biomarkers and Emerging Tools

Biomarkers are naturally occurring molecules that provide diagnostic value and add to the assessment of comorbidities [29,30]. Typical biomarkers include N-terminal pro-B-type natriuretic peptide, C-reactive protein, and cardiac troponins [20], which are often used to evaluate patients undergoing high-risk surgery [30].

While risk stratification tools are important in predicting postoperative outcomes and necessary preoperative optimization, their results are more tangible when they are also measured in patients. One study including 78 patients focused on the effects of adding biomarkers to ASA and ASC NSQIP in evaluating the risk stratification predictive value. Alone, ASA and ACS NSQIP had a reported area under the curve (AUC) of 0.669 and 0.813, respectively, and combined and AUC = 0.841 [31]. When both ASA and ASC NSQIP were combined with three biomarkers (baculoviral IAP repeat-containing protein 5 (BIRC5), heart-type fatty acid-binding protein (H-FABP), and high-sensitivity C-reactive protein (hsCRP)), the predictive value of the risk stratification increased (AUC = 0.941) compared to only hsCRP (AUC = 0.926) and without any biomarkers [17,31]. Similarly, when RCRI was used with biomarkers, the AUC was greater than RCRI alone [30,32]. In vascular surgery, high-sensitivity troponin testing showed a stronger association with better predictive value, with an AUC of 0.909 [33].

Biomarkers would have a stronger role in preoperative assessment if their addition to evaluation showed a strong association with adverse postoperative outcomes [29,34]. Future directions include optimizing existing risk stratification models by incorporating biomarkers to increase predictive value, developing machine learning algorithms that incorporate these biomarkers into risk assessments, and assessing interventions specific to biomarkers [29]. New biomarker research includes investigating inflammatory markers and cytokines and their role in postoperative complications; genetic and molecular (e.g., microRNA) markers to enable earlier detection of these events; and point-of-care testing to assess biomarkers closer to the time of surgery [29].

## 3. Preoperative Testing Modalities

### 3.1. Electrocardiography & Continuous Monitoring

Electrocardiography (ECG) is a 12-lead, inexpensive tool that can be used to evaluate a patient’s current cardiac status, as well as identify whether there have been past cardiovascular insults or unidentified cardiac conditions that require attention (e.g., arrhythmias) [14,35]. Past recommendations have recommended an ECG prior to noncardiac surgery (NCS) for evaluation of the patient preoperatively. Recently, specific scenarios have been included to help practitioners identify preoperative issues. Patient who have a history of genetic cardiomyopathy but are asymptomatic should have an ECG, those who are between the ages of 45–65 years with no symptoms of cardiac abnormalities should get an ECG prior to high risk NCS, and patients who have dyspnea or peripheral edema that is not explained by a noncardiac reason should be evaluated with an ECG before NCS [14].

Those presenting with chest pain in more urgent surgical situations may be screened with an ECG to exclude acute coronary syndrome. ECG is generally recommended to be obtained within 3 months of elective surgery [14,36]. This is to note any acute intraoperative changes not previously noted in the preoperative evaluation. Studies also indicate that postoperative ECG is recommended for patients with elevated N-terminal-pro-B-type natriuretic peptide (NT-proBNP) measurement before surgery or those who have an RCRI score > 1, age 45–64 with significant cardiovascular disease, or greater than 65 years old, to identify any acute cardiac changes immediately following surgery [15].

While the ECG provides information about a patient’s cardiac status in the moment, a more real-time assessment can be obtained with continuous cardiac monitoring. Postoperative atrial fibrillation (POAF) is a usual short-lasting response to surgical procedures seen mostly after cardiac cases, with an incidence of <3% after NCS [37]. After surgical intervention, factors contributing to POAF include increased sympathetic tone secondary to pain, hypovolemia, hypoxia, electrolyte abnormalities, hyperthyroidism, and the use of medications such as catecholamines [38,39]. Immediate recognition and treatment are important to prevent further complications such as stroke or mortality. Another study by Tian et al. showed that following bariatric surgery, continuous cardiac monitoring helped identify cardiovascular complications that would have gone undetected without enhanced postoperative monitoring strategies [40].

### 3.2. Non-Invasive Cardiac Imaging

Previous analyses of European and United States guidelines and studies have found low quality, high heterogeneity, and inconclusive evidence that non-invasive imaging should be performed preoperatively in patients with poor METs and at least 2 clinical risk factors for cardiac conditions [20]. In recent years, coronary computed tomography angiography (CCTA) has been adopted as an imaging modality that provides detailed morphologic information about coronary anatomy, making it a good screening tool for those with low-to-intermediate CAD risk [41].

With its high negative predictive value, clinicians have increased discrimination and perioperative risk stratification to better identify high-risk patients [42]. CCTA has been shown to be comparable to other non-invasive stress imaging, such as dobutamine stress echocardiography (DSE) and myocardial perfusion imaging (MPI) [41]. Furthermore, CCTA paired with RCRI has been shown to provide greater risk stratification than DSE alone; however, there is insufficient data on the anatomic (CCTA) versus ischemic (DSE and MPI) approach for perioperative ischemic assessment [41].

Studies also show heterogeneous data regarding the efficacy of stress tests in the preoperative evaluation of those with comorbidities [43]. Stress echocardiography has a high negative predictive value, but a low positive predictive value [44], which is useful in evaluating and managing patients with suspicious valvular heart disease or resting heart murmurs who are scheduled for intermediate or high-risk surgery [45,46]. Recently, more studies have aimed to demonstrate how stress echocardiograms may be used to better characterize valve abnormalities and the dynamic effects of these structural defects on overall function. Depending on the specific case, for instance, aortic stenosis with reduced versus preserved ejection fraction or primary versus chronic secondary mitral regurgitation, patients can be more specifically risk stratified in preoperative settings [47].

### 3.3. Invasive Testing

Along with non-invasive imaging methods, invasive imaging and testing are also used in a preoperative evaluation to assess risk. Guidelines note that invasive coronary angiography (ICA) is mostly indicated in patients undergoing non-urgent carotid endarterectomy who have stable cardiac conditions [20]. Routine pre-operative ICA is also not recommended in those who are undergoing low- to intermediate-risk NCS with stable cardiac history [14]. In patients with coronary artery disease, for instance, ICA is the gold standard for the evaluation of obstructive CAD [48]. Of note, the diagnostic potential of ICA has been reported at 38–50% in studies [48], and it is associated with higher complication rates, which add to health costs in the perioperative setting. Furthermore, ICA limits the study to the coronary vessels, whereas non-invasive CCTA also provides information on cardiac, pulmonary, and mediastinal structures, thereby enabling better perioperative planning [49].

## 4. Perioperative Medical Optimization

### 4.1. Antiplatelet Therapy

Perioperative medical optimization for patients on antiplatelet therapy necessitates a multifaceted approach to appropriately balance bleeding risk and ischemic events. Dynamic planning and decision making are of particular importance for patients with CAD and prior percutaneous coronary intervention (PCI) who are at increased risk of thrombosis. Current AHA/ACC guidelines for antiplatelet therapy recommend dual antiplatelet therapy (DAPT) with aspirin and clopidogrel for at least 6 months after PCI and for greater than 12 months in some circumstances after drug-eluting stent implantation or after myocardial infarction [50]. This makes timing a particularly important factor for noncardiac surgery in these patients.

The 2024 AHA/ACC guidelines recommend that patients with CAD undergoing elective noncardiac surgery should undergo multidisciplinary planning to minimize bleeding and thrombotic risk, including a risk/benefit analysis of delaying surgery [7]. As per these guidelines, it is recommended that elective noncardiac surgeries be delayed for patients who are within 14 days of coronary artery balloon angioplasty without stent placement. For noncardiac surgeries that require one or more antiplatelet therapies to be held, it is recommended that elective surgeries that are within 12 months of PCI with drug-eluting stent (DES) placement for acute coronary syndrome (ACS) be delayed if possible. Noncardiac surgery can be considered after 6 months from DES placement. Time-sensitive noncardiac surgery can be considered after 3 months of DES placement if the risk/benefit analysis favors surgery over the risk of perioperative major adverse cardiovascular events (MACE). Perioperative antiplatelet recommendations in patients who have had prior PCI include continuing 75–100 mg aspirin in all patients, continuing DAPT if patients have had bare-metal stents placed within 30 days or DES within 3 months, and bridging select high risk patients with intravenous antiplatelet therapy if within 6 months of DES placement and 30 days of bare-metal stent placement when noncardiac surgery cannot be delayed.

A 2018 meta-analysis exploring perioperative bleeding risk in noncardiac surgery with use of aspirin monotherapy, clopidogrel monotherapy, and dual antiplatelet therapy found bleeding risk (measured by risk of requiring transfusion) increased stepwise from aspirin to clopidogrel, with dual antiplatelet therapy being the highest risk [51]. However, while bleeding risk was elevated in these groups, the need for reintervention for bleeding control was not statistically increased. This suggests that continuing antiplatelet therapy is a potential option for many of the high-risk patients previously discussed.

### 4.2. Statins and Risk Modification

Given that many of these patients would also be on statin therapy, it is also important to establish guidelines in perioperative statin therapy management. Perioperative statin use has been established to be safe in both statin-naïve patients and patients already on statin therapy [7]. Some studies have suggested that perioperative statin use can even decrease perioperative cardiovascular complications in noncardiac surgeries [52]. However, the Lowering the Risk of Operative Complications Using Atorvastatin Loading Dose (LOAD) trial, the largest RCT on perioperative statin use, found no difference in perioperative MACE among statin-naïve patients who received perioperative atorvastatin compared with the placebo [53]. While it is still unclear whether perioperative statin use provides short-term benefit in noncardiac surgery, it has been established as safe to continue statins and even start statin therapy for statin-naïve patients in some scenarios [7]. The 2024 AHA/ACC guidelines recommend that for patients already on a statin, statin therapy should be continued for noncardiac surgery. For statin-naïve patients undergoing noncardiac surgery, statin initiation can be determined based on the 2013 ASCVD guidelines and can be continued for long-term use.

### 4.3. Beta-Blockers & ACE Inhibitors

Perioperative management of beta-blockers and renin–angiotensin–aldosterone system inhibitors is more complex than that of statin therapy. Studies have found that starting beta-blockers perioperatively could decrease nonfatal myocardial infarction but also found an increase in stroke, death, bradycardia, and hypotension, and is therefore not recommended [53]. Therefore, the 2024 AHA/ACC guidelines recommend against starting beta-blockers on the day of surgery [7]. For patients with a new indication for beta-blockers undergoing noncardiac surgery, it is recommended to start beta-blocker therapy more than seven days prior to surgery to allow for dosage titration and optimization. Patients on a stable beta-blocker dose who are undergoing noncardiac surgery are recommended to continue beta-blockers perioperatively if appropriate.

Patients with no acute need for beta-blocker therapy should not be started on one during the operative period. Data regarding perioperative management of renin–angiotensin–aldosterone system inhibitors is still limited. Currently, it is recommended that renin–angiotensin–aldosterone system inhibitors be held 24 h preoperatively in patients with controlled blood pressures undergoing high-risk procedures [7]. For patients with heart failure with reduced ejection fraction, continuing renin–angiotensin–aldosterone system inhibitors can be considered.

## 5. Integration of 2024 AHA/ACC Guidelines

### 5.1. Key New Recommendations

Notable new recommendations in the 2024 AHA/ACC guidelines include the use of cardiac biomarkers for preoperative risk stratification, updates to preoperative diagnostic testing, guidelines for perioperative management of new-onset atrial fibrillation, new perioperative medication guidelines, and more specific recommendations for noncardiac surgery timing regarding PCI, and MINS surveillance [7].

- The use of B-type natriuretic peptide (BNP), NT-proBNP, and cardiac troponin measurements for preoperative risk stratification in patients with cardiovascular disease (CVD), older than 65 years, or older than 45 years and CVD symptoms for elevated risk noncardiac surgery.
- The use of CCTA to detect coronary abnormalities in select high-risk patients.
- For patients with new-onset perioperative/postoperative atrial fibrillation, recommendations include the management of provoking factors such as pain, anemia, sepsis, electrolyte disturbances, and fluid shifts, and rhythm control to a goal heart rate under 110 bpm.
- Recommendations regarding perioperative medication management now include stopping sodium-glucose cotransporter-2 inhibitors 3–4 days prior to noncardiac surgery and 4+ days for ertugliflozin specifically.
- More detailed recommendations regarding the timing of noncardiac surgeries after PCI were made, as discussed in Section 4.1.
- Cardiac troponin surveillance may be reasonable in patients with known cardiovascular disease, cardiovascular risk factors, or those undergoing higher-risk noncardiac surgery to identify myocardial injury postoperatively at 24 and 48 h.

### 5.2. Special Populations

Special populations discussed in the 2024 AHA/ACC guidelines are patients with obesity and end-stage kidney and liver disease patients requiring transplantation [7]. Cardiac screening and surveillance for kidney transplant patients vary widely across kidney transplant programs in the United States [54]. More recent literature suggests that enhanced cardiac screening or intervention in end-stage kidney disease patients beyond what is routine does not lead to better outcomes and may not outweigh the costs and possible harm [7,55]. Similarly, liver transplantation programs in the United States also vary in cardiac screening protocols [56]. The 2022 AHA statement on kidney and liver transplant candidates provides possible approaches to the screening and management of these two special populations [55]. An established clinical algorithm is not yet available, but a proposed approach recommends kidney and liver transplant candidates with symptoms of cardiac disease to have cardiology referral and be managed as per the ACC/AHA guidelines. Kidney and liver transplant candidates without symptomatic cardiac disease or known coronary heart disease are recommended to get screened with a transthoracic echocardiogram and EKG and be managed based on those results. With the growing incidence of obesity, bariatric surgery patients and patients on glucagon-like peptide-1 (GLP-1) receptor agonists for weight loss are additional special populations mentioned in the 2024 AHA/ACC guidelines [55]. It is recommended that attention be given to the preoperative assessment of these patients, given the risk of bariatric surgery and high chance of significant cardiac history. Additionally, considerations need to be made for these patients if they are on GLP-1 receptor agonists. The 2024 AHA/ACC guidelines refer to the ASA recommendations of holding GLP-1 agonists for greater than one week before NCS for weekly dosed drugs and one day for daily dosed drugs to reduce aspiration risk [7]. However, more recent studies suggest that the management of GLP-1 agonists in the perioperative period should be based on individual patient risk rather than routinely holding these medications for all patients [57].

## 6. Perioperative Ischemic Event Management Pathways

### 6.1. Early Recognition & Monitoring Protocols

MINS can be difficult to recognize because most events occur without typical ischemic symptoms of chest pain or dyspnea. Also, electrocardiographic abnormalities are frequently absent [58]. This atypical presentation may be due to the masking effects of sedation, analgesia, and physiologic stress following surgery. Monitoring strategies, therefore, increasingly rely on postoperative cardiac troponin surveillance. The 2024 AHA/ACC guidelines note that troponin surveillance may be reasonable in patients with known cardiovascular disease, cardiovascular risk factors, or those undergoing higher-risk noncardiac surgery [7]. In practice, troponin is measured within the first 24–48 h after surgery in higher-risk patients, when most perioperative myocardial injuries occur [3,58].

MINS is defined as at least one postoperative cardiac troponin elevation about the 99th percentile upper reference limit attributable to presumed ischemia [58]. In a representative cohort of surgical inpatients ≥ 45 years of age undergoing noncardiac surgery, the number needed to screen to detect otherwise-missed myocardial injury is approximately 15 patients [59]. A baseline troponin level obtained before surgery can help distinguish new perioperative elevations from chronically elevated values. Postoperative troponin elevation is strongly associated with adverse outcomes, with approximately 10% 30-day mortality [3]. Despite the ability of troponin surveillance to identify high-risk patients, the best approach to management for MINS patients is still unclear, as randomized clinical trials investigating possible treatment methods are limited [58,60].

### 6.2. Algorithms for Acute Management

A structured approach is necessary for evaluating and managing perioperative myocardial injury. Initial assessment categorizes injury into ischemic versus nonischemic causes of troponin elevation, such as sepsis, pulmonary embolism, and acute heart failure. Management focuses on correcting reversible causes of increased myocardial oxygen supply–demand imbalance, such as hypotension, anemia, hypoxemia, and tachyarrhythmias. If the ACS criteria are met, treatment generally follows the established ST-segment elevation and non-ST-segment elevation myocardial infarction management pathways while accounting for perioperative bleeding risk [7]. For patients with MINS who do not meet the ACS criteria, cardiovascular follow-up is recommended. A recent multicenter prospective study of over 14,000 high-risk surgical patients found that cardiologist evaluation following MINS was independently associated with a nearly 50% reduction in major adverse cardiac events at 1 year [61]. However, the observational design is subject to confounding by indication, as patients evaluated by a cardiologist may have differed systemically from those who were not evaluated. These findings support the consideration of early multidisciplinary involvement in the management of MINS.

### 6.3. Quality Improvement and Safety Measures

System-level strategies may improve early recognition and the management of MINS. However, studies suggest that MINS often goes underrecognized and undertreated in routine practice. In a prospective single-center study, only approximately half of the patients with MINS were evaluated by cardiology consultants, few underwent additional cardiac testing, and only 29% received intensification of guideline-directed cardiovascular therapy [58]. These data illustrate a gap between guideline recommendations and clinical practice. Resolving this gap will likely require practical institutional changes such as standardized troponin surveillance protocols, postoperative checklists, and electronic health record alerts. In addition, closer collaboration among surgical, anesthesiology, and cardiology teams may facilitate timely follow-up and the optimization of cardiovascular therapy.

## 7. Discussion

### 7.1. Interpreting the Evidence Base

The contemporary approach to perioperative ischemic workup illustrates a change from earlier strategies that emphasized routine diagnostic testing toward a more selective, evidence-based strategy. A consistent finding across multiple large-scale studies analyzed in this review is that perioperative myocardial injury is common, frequently clinically silent, and strongly associated with adverse outcomes. Observational findings from the VISION cohort and related studies demonstrated that postoperative troponin elevation (even in the absence of ischemic symptoms) is independently associated with increased 30-day and long-term mortality [3,4,5]. These conclusions established MINS as a clinically meaningful entity and shifted the focus of perioperative cardiac evaluation beyond overt myocardial infarction.

Despite the strength of this prognostic association, interventional evidence remains limited. Randomized trials such as the CARP trial demonstrated no benefit of routine preoperative coronary revascularization in stable patients undergoing vascular surgery [12]. Similarly, trials evaluating perioperative pharmacologic interventions have yielded mixed results. The Perioperative Ischemic Evaluation (POISE) trial demonstrated a reduction in myocardial infarction with perioperative beta-blockade, but at the cost of increased stroke and mortality [62], while the POISE-2 trial showed that perioperative aspirin did not reduce cardiovascular events but increased the bleeding risk [63]. Collectively, these findings reinforce that reducing ischemic risk is not only dependent on identifying coronary anatomy, but rather on managing the complex physiologic stresses of surgery.

The 2024 AHA/ACC guidelines synthesize these data into a pragmatic framework emphasizing clinical risk stratification, functional capacity, and selective testing [7,8]. Importantly, the guidelines reiterate that diagnostic testing should only be pursued when it is expected to change management. This demonstrates a broader shift toward value-based care and avoidance of low-yield interventions.

Emerging evidence also supports integrating biomarkers into perioperative risk assessment. Preoperative natriuretic peptides and postoperative high-sensitivity troponin measurements have demonstrated strong associations with adverse cardiovascular outcomes [6,16]. However, while these biomarkers enhance risk prediction, their role in guiding therapeutic decision-making is still under investigation.

### 7.2. Clinical Implementation Challenges

Despite increasingly clear evidence and guideline recommendations, implementing these principles in routine clinical practice presents challenges. Perioperative care is necessarily multidisciplinary, involving anesthesiologists, surgeons, cardiologists, and internists, which creates variability in clinical decision-making. Risk stratification tools such as the RCRI and NSQIP are widely available but inconsistently applied, and their predictive effectiveness varies depending on the surgical population and patient characteristics [10,14,64]. Assessment of functional capacity presents an additional barrier. While subjective estimation continues to be common, studies have demonstrated that clinician-assessed functional capacity is relatively unreliable compared to structured tools such as the DASI scores or CPET [26,27]. However, CPET is costly and not universally accessible, limiting its broad adoption.

Again, the incorporation of biomarkers into clinical processes remains controversial. Although natriuretic peptides and troponin improve risk prediction, clinicians often face uncertainty about how abnormal values should influence management decisions. For example, elevated preoperative BNP or NT-proBNP identifies higher-risk patients but does not necessarily support further testing or surgical delay [15,16]. Similarly, postoperative troponin surveillance can detect MINS, yet optimal treatment strategies are still uncertain due to limited randomized data [60].

Another important challenge is balancing comprehensive evaluation with surgical urgency. In urgent or emergent procedures, there may be insufficient opportunity for an extensive cardiac workup. In these cases, rapid risk stratification and intraoperative management strategies are utilized instead of formal testing algorithms. Finally, healthcare system factors (including cost, resource availability, and institutional practice policies) may contribute to either the overuse or underuse of diagnostic testing. For example, Wijeysundera et al. showed that preoperative stress testing is frequently performed despite lacking clear indications and may not improve outcomes when used indiscriminately [11].

### 7.3. Risk–Benefit Balancing

The process of performing a perioperative ischemic evaluation is largely defined by balancing the risks of cardiovascular complications against the risks associated with testing, process delays, and clinical interventions. Evidence repeatedly demonstrates that more testing does not necessarily lead to better outcomes. Routine stress testing or invasive coronary angiography in stable patients may identify coronary disease, but rarely changes management in a way that improves perioperative outcomes [11,12].

Perioperative medication management further illustrates this balance. Ongoing administration of antiplatelet therapy may reduce thrombotic risk but increase bleeding risk, particularly in high-risk surgical procedures [51]. Similarly, while beta-blockers may reduce myocardial oxygen demand, inappropriate initiation or dosing can increase the risk of hypotension, stroke, and mortality [62]. Current AHA/ACC guidelines therefore emphasize maintaining chronic therapies and cautiously initiating new therapies well in advance of surgery [7]. Biomarker-guided strategies are a promising but developing approach to improving risk–benefit decision-making. Elevated troponin or natriuretic peptide levels may identify patients who would benefit from closer monitoring or cardiology involvement. Notably, recent data suggest that cardiology consultation following MINS is associated with improved outcomes, supporting the value of multidisciplinary care [61].

**Figure 1:**
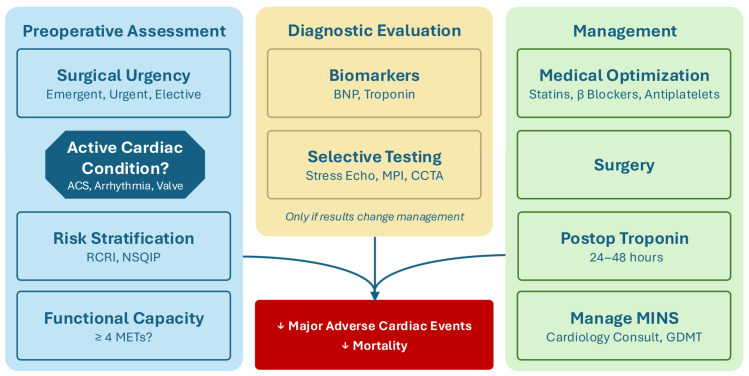
Current perioperative ischemic evaluation/management considerations.

Ultimately, optimal perioperative ischemic workup requires individualized, patient-centered decision-making. This begins with perioperative assessment, continues with diagnostic evaluation, and concludes with management. See Figure 1 for an original schematic synthesized from the findings of this narrative review. Rather than maximizing diagnostic testing, guidelines emphasize evaluation and management with the patient’s overall risk profile, surgical urgency, and likelihood of benefit from intervention.

## 8. Conclusions

Perioperative myocardial ischemia and myocardial injury after noncardiac surgery remain major contributors to postoperative morbidity and mortality. Over the past two decades, the recognition of MINS and the widespread use of high-sensitivity cardiac biomarkers have fundamentally changed the understanding of perioperative cardiovascular risk [3,6]. Contemporary perioperative ischemic workup, as outlined in the 2024 AHA/ACC guidelines, emphasizes a structured, stepwise approach including clinical risk assessment, functional capacity evaluation, and selective diagnostic testing [7,8]. This approach favors interventions that meaningfully impact management while limiting unnecessary testing and associated harms.

Even with these advances, important gaps remain. Variability in implementation, limited interventional evidence for MINS management, and uncertainty regarding biomarker-guided strategies demonstrate the need for further research. Future directions consist of refining risk prediction models, integrating biomarkers into decision-making pathways, and enhancing multidisciplinary perioperative care. A balanced, individualized approach, grounded in evidence, clinical judgment, and guideline-directed care, remains essential for optimizing perioperative cardiovascular outcomes in an increasingly complex surgical population.

## 9. Future Directions

### 9.1. Knowledge Gaps Identified

Although there is increasing clinical awareness of MINS, several knowledge gaps remain. While postoperative cardiac troponin surveillance reliably identifies patients with increased risk of MINS, there is limited guidance for management [7]. Most current evidence on management is empirical, with few randomized trials investigating specific treatment strategies. The Management of Myocardial Injury after Noncardiac Surgery (MANAGE) trial randomized 1754 patients ≥ 45 years of age with MINS of presumed ischemic origin to dabigatran or placebo. At a mean follow-up of 16 months, dabigatran reduced the primary composite outcome of major vascular complications (11% vs. 15%; HR 0.72; 95% CI, 0.55–0.93), although further studies are needed to define the role of antithrombotic therapy and other cardiovascular interventions [58]. Also, in prospective studies, fewer than one-third of patients with MINS had an intensification of guideline-directed cardiovascular medical therapy [7]. This suggests a gap between detection and clinical action.

### 9.2. Research Priorities

Several areas warrant further investigation. Randomized controlled trials are needed to determine whether treatments such as antithrombotic therapy, cardiovascular medical therapy optimization, and perioperative hemodynamic management improve clinical outcomes in patients with MINS. Another area of future study is refining patient selection for surveillance programs. Risk prediction models and biomarker-guided approaches may help identify patients most likely to benefit from surveillance. Finally, implementation research is needed to overcome the persistent gaps between guideline recommendations and everyday practice. Studies evaluating standardized troponin surveillance pathways, multidisciplinary care models, and structured outpatient follow-up may help improve the recognition and management of perioperative myocardial injury.

### 9.3. Toward Precision Perioperative Ischemic Care

Advances in predictive analytics and precision medicine may help improve perioperative cardiovascular risk assessment. Machine learning-based models that integrate clinical, laboratory, and intraoperative variables have demonstrated better discrimination for predicting MINS compared with traditional clinical risk scores. Several internally validated machine learning models have reported areas under the receiver operating characteristic curve of approximately 0.77–0.78, suggesting improved discrimination compared with traditional clinical risk scores, although external validation is needed to establish their generalizability [65,66].

Genomic approaches may also offer a more individualized understanding of perioperative ischemic risk. Polygenic risk scores for CAD have been associated with MINS, although improvements in predictive performance remain modest [67]. Recent analyses from the VISION cohort suggest that genetic markers associated with glycemic regulation may also be linked to MINS, highlighting potential biological pathways warranting further investigation [68].

## Figures and Tables

**Table 1:** Overview of select surgical risk calculator tools and notable characteristics of each.

	Variables	Scores	Evaluated Outcomes	Advantages	Limitations
RCRI	High risk surgery classification; History of IHD ^1^; History of CHF ^2^; History of CVD ^3^; Preoperative treatment with insulin; Preoperative creatinine > 2 mg/dL	0–6	Postoperative cardiac outcomes (noncardiac surgery)	Identifies at-risk patients preoperatively	Uncertain generalizability and lacks objective metrics
NSQIP	Age; Sex; Functional Status; Urgency of Surgery; ASA ^4^ Class; Immunosuppressive therapy; Ascites within 30 days prior to surgery; Systemic sepsis within 48 h prior to surgery; Ventilatory dependent; Disseminated cancer; Diabetes, Hypertension requiring medications; CHF 30 days prior to surgery; Oxygen support; Smoking history within the year; COPD; Dialysis; Stage 2/3 acute kidney failure	C-statistic 0.0–1.0	30-day mortality and morbidity risk	Detailed analysis of specific risks	Not generalizable outside of USA
SORT	Age; ASA physical status; Surgical urgency; Surgical specialty; Surgical severity; Malignancy Status	Percentage risk of mortality	30-day mortality risk	Compares emergent and elective surgeries	Does not examine morbidity risks
ARISCAT	Age; Preoperative SpO_2_; Respiratory infection in the past month; Preoperative anemia; Surgical incision location; Duration of surgery; Surgical Urgency	0–123	Postoperative pulmonary complications	Identifies at risk patients preoperatively	No consensus on specific postoperative complications to focus on
Gupta Perioperative Risk for Myocardial Infarction or Cardiac Arrest	Age; Functional Status; ASA Class; Creatinine; Type of Procedure	Calculated risk percentage by formula	Risk of myocardial infarction or cardiac arrest, intraoperatively or postoperatively	More targeted cardiac preoperative assessment in high-risk cases	Does not assess beyond myocardial infarction and cardiac death

^1^ Ischemic heart disease ^2^ Congestive heart failure ^3^ Cerebrovascular disease ^4^ American Society of Anesthesiologists.

## Data Availability

The original data presented in the study are openly available in PubMed at https://pubmed.ncbi.nlm.nih.gov.

## Abbreviations

The following abbreviations are used in this manuscript:

MINS	Myocardial Injury After Noncardiac Surgery
CAD	Coronary Artery Disease
VISION	Vascular Events In Noncardiac Surgery Cohort Evaluation
RCRI	Revised Cardiac Risk Index
CARP	Coronary Artery Revascularization Prophylaxis
AHA/ACC	American Heart Association/American College of Cardiology
NSQIP	National Surgical Quality Improvement Program
SORT	Surgical Outcome Risk Tool
MET	Metabolic Equivalent
DASI	Duke Activity Status Index
CPET	Cardiopulmonary Exercise Testing
AUC	Area Under the Curve
ECG	Electrocardiography
NT-proBNP	N-Terminal-Pro-B-Type Natriuretic Peptide
POAF	Postoperative Atrial Fibrillation
NCS	Noncardiac Surgery
CCTA	Coronary Computed Tomography Angiography
DSE	Dobutamine Stress Echocardiography
MPI	Myocardial Perfusion Imaging
ICA	Invasive Coronary Angiography
ARISCAT	Assess Respiratory Risk In Surgical Patients In Catalonia Tool
DAPT	Dual Antiplatelet Therapy
PCI	Percutaneous Coronary Intervention
DES	Drug-Eluting Stent
ACS	Acute Coronary Syndrome
MACE	Major Adverse Cardiovascular Events
LOAD	Lowering the Risk of Operative Complications Using Atorvastatin Loading Dose
ASCVD	Atherosclerotic Cardiovascular Disease
BNP	B-Type Natriuretic Peptide
CVD	Cardiovascular Disease
GLP-1	Glucagon-Like Peptide-1
POISE	Perioperative Ischemic Evaluation
MANAGE	Management of Myocardial Injury After Noncardiac Surgery
